# Too much control diverts from the essence of learning and teaching

**DOI:** 10.1007/s40037-015-0216-6

**Published:** 2015-09-10

**Authors:** Anneke W.M. Kramer

**Affiliations:** Department of Public Health and Primary Care, Leiden University Medical Center, PO Box 9600, 2300 RC Leiden, The Netherlands

To the editor,

In the last ten years, interest in competency-based medical education (CBME) has gathered momentum [1]. This relatively new approach towards medical education originated from a need for more accountability and quality assurance and for a greater emphasis on curriculum aspects that were previously obscured. Its aim was to increase transparency, so that the profession and the public could rest assured that medical education would deliver competent physicians who were equipped with knowledge and skills that fitted practice. Its introduction, however, has gone hand in hand with zealous attempts to specify outcomes, content and assessment of medical education (roles, competencies, entrustable professional activities, milestones, etc.). In Canada, for instance, the new draft version of the 2015 CanMEDS contains, in addition to the roles and competencies, 710 milestones that lay down the whole medical education continuum from day 1 of undergraduate school through to continuing professional development [2]. Also speciality training for general practice in the Netherlands recently introduced 82 key professional activities [3]. All of these efforts drain a lot of energy, time, and money, not to mention what it will take to implement these stipulations and how this will affect trainees and their teachers. It would all be worth the effort if it were lead to better doctors. However, I have some serious concerns about this, because I think this zeal for structuring diverts from the essence of learning. Learning is not only about acquiring knowledge and skills but, above all and especially in the workplace, it is about participating in practice and lending meaning to experiences with the aid of supervisors [4]. Workplaces are messy environments in which learners have to deal with whatever crops up. To determine in advance what should be taught and learned in practice is to ignore the unpredictability of the practice and the sudden presentation of new situations in which you are forced to find out by trial and error how best to behave professionally. It is in this context that learners should become professionals. What has to be learned, then, is context-dependent and personal; the outcome cannot be described in well-defined criteria but should instead be more reflective and process-oriented. By limiting medical education to narrowly defined content and outcome we ignore the very nature of medical practice which features uncertainty and ambiguity. This ambiguity and uncertainty is precisely what future doctors must learn to deal with [5]. In this letter I will express my dissent from this trend of detailing medical education content, based on my personal experiences and the literature.

In 1981, I started my residency in general practice in the Netherlands. This residency had been launched a few years earlier without detailed descriptions of learning outcomes. Guiding principles of this training programme were *The Basic Job Description of the General Practitioner*, drawn up by the professional body, and educational insights, for instance about the importance of supervised workplace-based learning, time for reflection, formal learning, and meeting peers. I learned a lot, especially in the workplace, but also from the weekly peer meetings that were facilitated by a general practitioner and a social sciences teacher. Precisely because there was no programme detailing all the specifics, we felt encouraged to self-direct our learning. As a result, we engaged ourselves in searching for evidence, developing our communication skills, discussing existential experiences and reflecting on what was needed to become good doctors. I am of the opinion that I was not a bad general practitioner at the start of my practice, and that I am still sufficiently competent this very day. Perhaps the life-long learning skills I believe I possess were acquired in residency training, yet, I feel the need to point out that medical practice forces me to keep up. I know from experience that a global description of the course content, together with supervised learning in practice, and time for deliberate learning supported by teachers, give enough guidance to become and stay a sufficiently competent doctor; I also learned that continuous practice can be a powerful motor that drives life-long learning.

Literature provides ample evidence that, because of societal and scientific developments, both medicine and medical education are in constant movement [6]. Current developments are inspired by a call for more transparency and accountability on the one hand, and a plea for self-regulated and transformative education, characterized by diversity and unpredictability, on the other [1, 7, 8]. The challenge for medical education is to strike a balance between these seemingly opposing requirements. With the introduction of competencies, few in number and generally described, it seemed that a simple solution had been found to achieve greater transparency while also accommodating diversity and unpredictability. However, over the years this simplicity has rapidly gotten lost in a continuous process of specification [1–3]. Apart from the difficulty of conceptualization this approach presents, it leads to problems of assessment (how to assess an unrealistic sample of observations; creation of tick-box exercises), logistics (how to implement), and an administrative burden (having to report all this information) [1, 9].

What’s more, essential attributes of learners and teachers are that they be intrinsically motivated, inspired, and have a sense of ownership [8, 10]. Too much interference with workplace-based learning (e.g. by administering competency-based assignments), and too much bureaucracy (e.g. by making portfolios a compulsory component) causes frustration and demotivates, and diverts from the primary process of learning in practice [8, 11]. In view of the societal and educational call for self-regulating and transformative professionals, medical education should encourage learners to take responsibility for their own learning and empower them to cope with tremendous societal changes, such as ageing and the explosion of knowledge and costs, by teaching them how they can help transform the health care system accordingly. This is also why the leadership role is receiving so much attention [12]. If we want doctors who feel responsible for the quality of their performance and for the health care system in which they operate, we need an approach that teaches learners to take responsibility not only for learning the formal requirements, but also for *what* needs to be learned from informal practice and *how* this should be learned. As self-regulated learning is a skill that must be acquired first and teachers and supervisors play an important role therein, their focus should not exclusively be on content but also on how to cultivate self-directed learning [11]. In this regard too, research provides ample evidence that too much external involvement disturbs the delicate process whereby, with balanced support and entrustment from the supervisor, trainees become self-regulated learners [11]. Hence, to recap, in my view we put too much emphasis on structuring the content of medical education, which comes at the expense of the learning process. The workplace offers a very powerful learning environment for becoming a professional, therefore we should pay more attention to how to support this learning in practice in order to deliver good doctors who can deal with ambiguity and uncertainty and are life-long learners and change agents.
